# Supplementary material for: Identification of HMGA2 inhibitors by AlphaScreen-based ultra-high-throughput screening assays
Source: Sci Rep. 2020 Nov 2;10:18850. doi: 10.1038/s41598-020-75890-0 (PMC7606612; doi:10.1038/s41598-020-75890-0)
Supplement: Supplementary file 1 — Supplementary Information. [file 41598_2020_75890_MOESM1_ESM.pptx]

## Slide 1
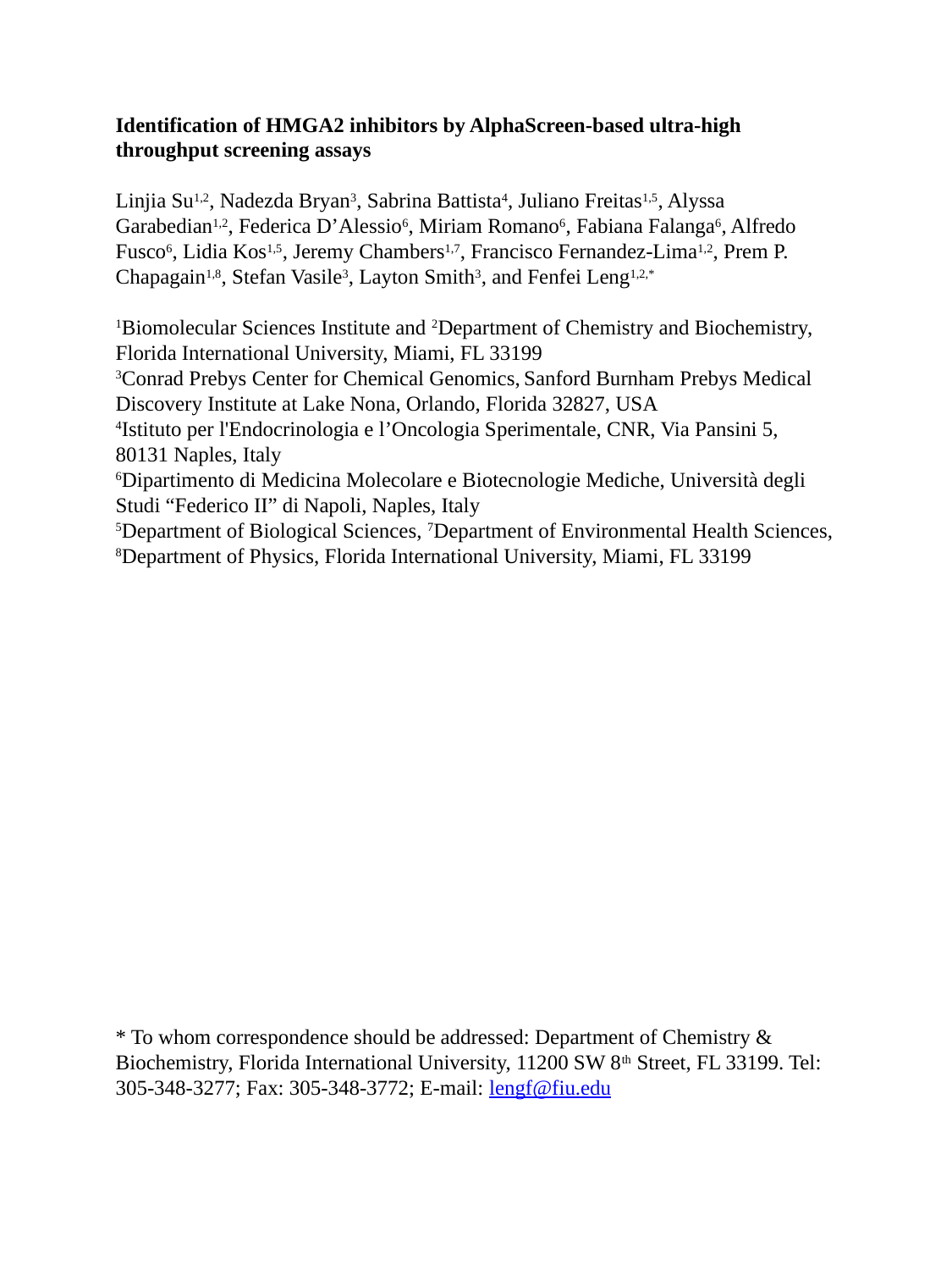

Identification of HMGA2 inhibitors by AlphaScreen-based ultra-high throughput screening assays
Linjia Su1,2, Nadezda Bryan3, Sabrina Battista4, Juliano Freitas1,5, Alyssa Garabedian1,2, Federica D’Alessio6, Miriam Romano6, Fabiana Falanga6, Alfredo Fusco6, Lidia Kos1,5, Jeremy Chambers1,7, Francisco Fernandez-Lima1,2, Prem P. Chapagain1,8, Stefan Vasile3, Layton Smith3, and Fenfei Leng1,2,*
1Biomolecular Sciences Institute and 2Department of Chemistry and Biochemistry, Florida International University, Miami, FL 33199
3Conrad Prebys Center for Chemical Genomics, Sanford Burnham Prebys Medical Discovery Institute at Lake Nona, Orlando, Florida 32827, USA
4Istituto per l'Endocrinologia e l’Oncologia Sperimentale, CNR, Via Pansini 5, 80131 Naples, Italy
6Dipartimento di Medicina Molecolare e Biotecnologie Mediche, Università degli Studi “Federico II” di Napoli, Naples, Italy
5Department of Biological Sciences, 7Department of Environmental Health Sciences, 8Department of Physics, Florida International University, Miami, FL 33199
* To whom correspondence should be addressed: Department of Chemistry & Biochemistry, Florida International University, 11200 SW 8th Street, FL 33199. Tel: 305-348-3277; Fax: 305-348-3772; E-mail: lengf@fiu.edu

## Slide 2
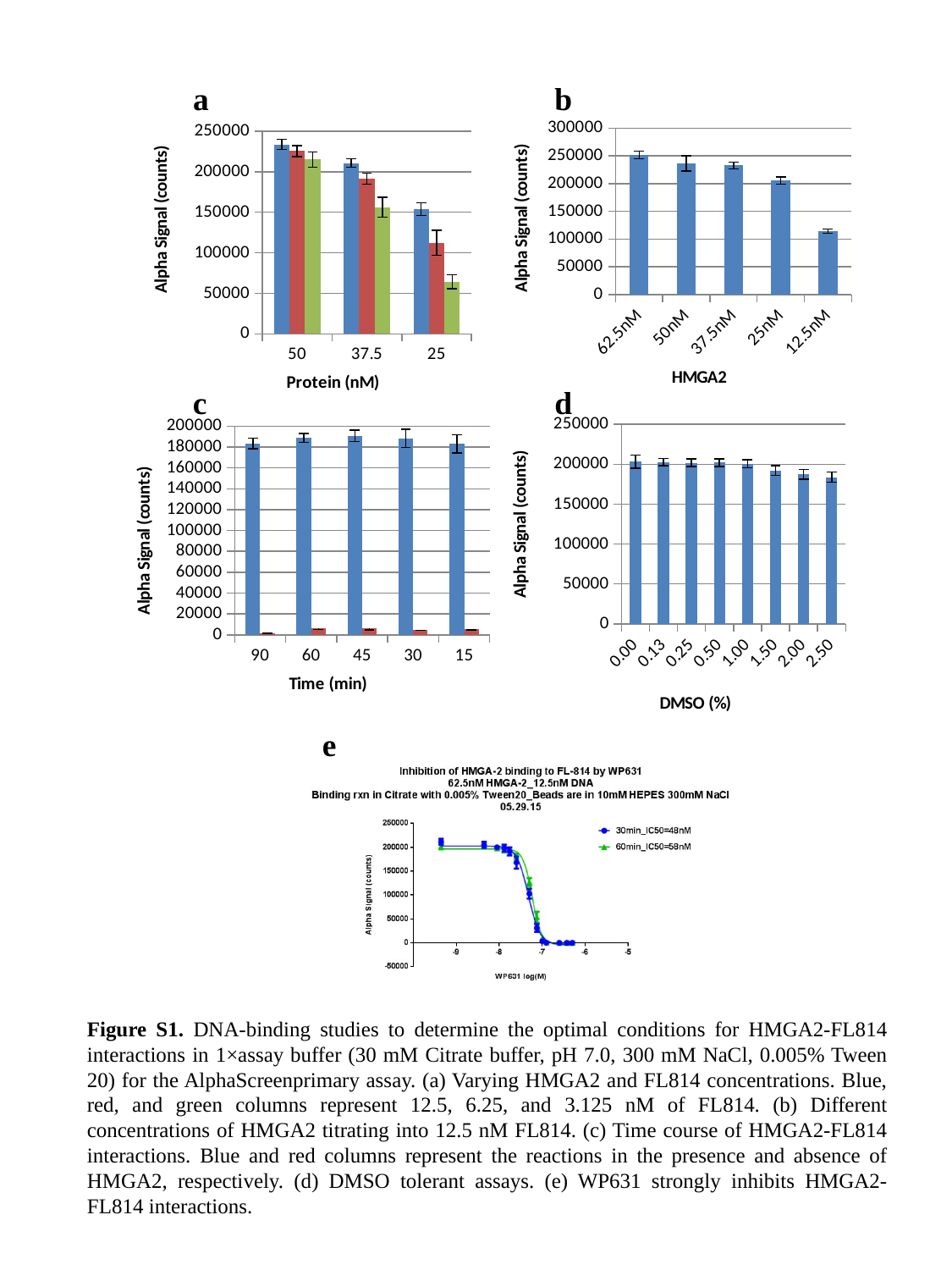

a
b
### Chart
| Category | |
|---|---|
| 62.5nM | 251772.7333333333 |
| 50nM | 236120.775 |
| 37.5nM | 232543.425 |
| 25nM | 205354.025 |
| 12.5nM | 114586.5 |
### Chart
| Category | | | |
|---|---|---|---|
| 50.0 | 233517.8095238095 | 225435.2857142857 | 214915.0 |
| 37.5 | 210656.8333333333 | 191425.84375 | 156314.59375 |
| 25.0 | 153957.1666666667 | 112297.34375 | 64318.5625 |c
d
### Chart
| Category | |
|---|---|
| 0.0 | 203319.9677419355 |
| 0.125 | 202786.1451612903 |
| 0.25 | 201845.6774193548 |
| 0.5 | 202115.6451612903 |
| 1.0 | 200717.6129032258 |
| 1.5 | 192284.8548387096 |
| 2.0 | 187415.6129032258 |
| 2.5 | 183877.629032258 |
### Chart
| Category | | |
|---|---|---|
| 90.0 | 183342.9569892473 | 1743.225806451613 |
| 60.0 | 188586.6344086021 | 5903.419354838707 |
| 45.0 | 190740.8709677419 | 5974.032258064517 |
| 30.0 | 188441.0107526882 | 5001.354838709678 |
| 15.0 | 182915.752688172 | 5859.61290322581 |e
Figure S1. DNA-binding studies to determine the optimal conditions for HMGA2-FL814 interactions in 1×assay buffer (30 mM Citrate buffer, pH 7.0, 300 mM NaCl, 0.005% Tween 20) for the AlphaScreenprimary assay. (a) Varying HMGA2 and FL814 concentrations. Blue, red, and green columns represent 12.5, 6.25, and 3.125 nM of FL814. (b) Different concentrations of HMGA2 titrating into 12.5 nM FL814. (c) Time course of HMGA2-FL814 interactions. Blue and red columns represent the reactions in the presence and absence of HMGA2, respectively. (d) DMSO tolerant assays. (e) WP631 strongly inhibits HMGA2-FL814 interactions.

## Slide 3
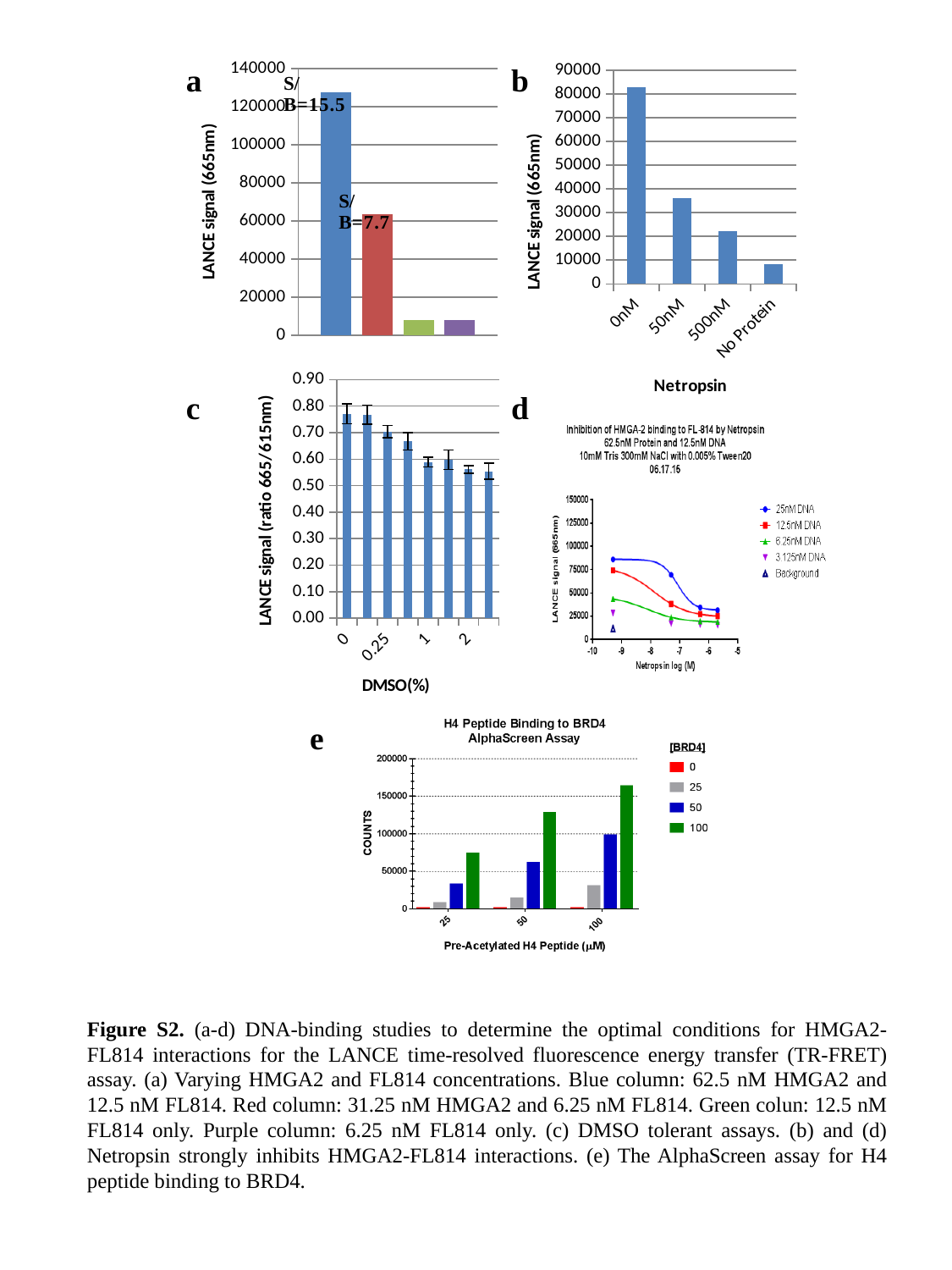

### Chart
| Category | | | | |
|---|---|---|---|---|a
### Chart
| Category | |
|---|---|
| 0nM | 82913.0 |
| 50nM | 36272.0 |
| 500nM | 22085.0 |
| No Protein | 8214.0 |b
### Chart
| Category | |
|---|---|
| 0.0 | 0.770925 |
| 0.125 | 0.767625 |
| 0.25 | 0.704275 |
| 0.5 | 0.6671 |
| 1.0 | 0.588425 |
| 1.5 | 0.5972 |
| 2.0 | 0.56165 |
| 2.5 | 0.5544 |c
d
e
Figure S2. (a-d) DNA-binding studies to determine the optimal conditions for HMGA2-FL814 interactions for the LANCE time-resolved fluorescence energy transfer (TR-FRET) assay. (a) Varying HMGA2 and FL814 concentrations. Blue column: 62.5 nM HMGA2 and 12.5 nM FL814. Red column: 31.25 nM HMGA2 and 6.25 nM FL814. Green colun: 12.5 nM FL814 only. Purple column: 6.25 nM FL814 only. (c) DMSO tolerant assays. (b) and (d) Netropsin strongly inhibits HMGA2-FL814 interactions. (e) The AlphaScreen assay for H4 peptide binding to BRD4.

## Slide 4
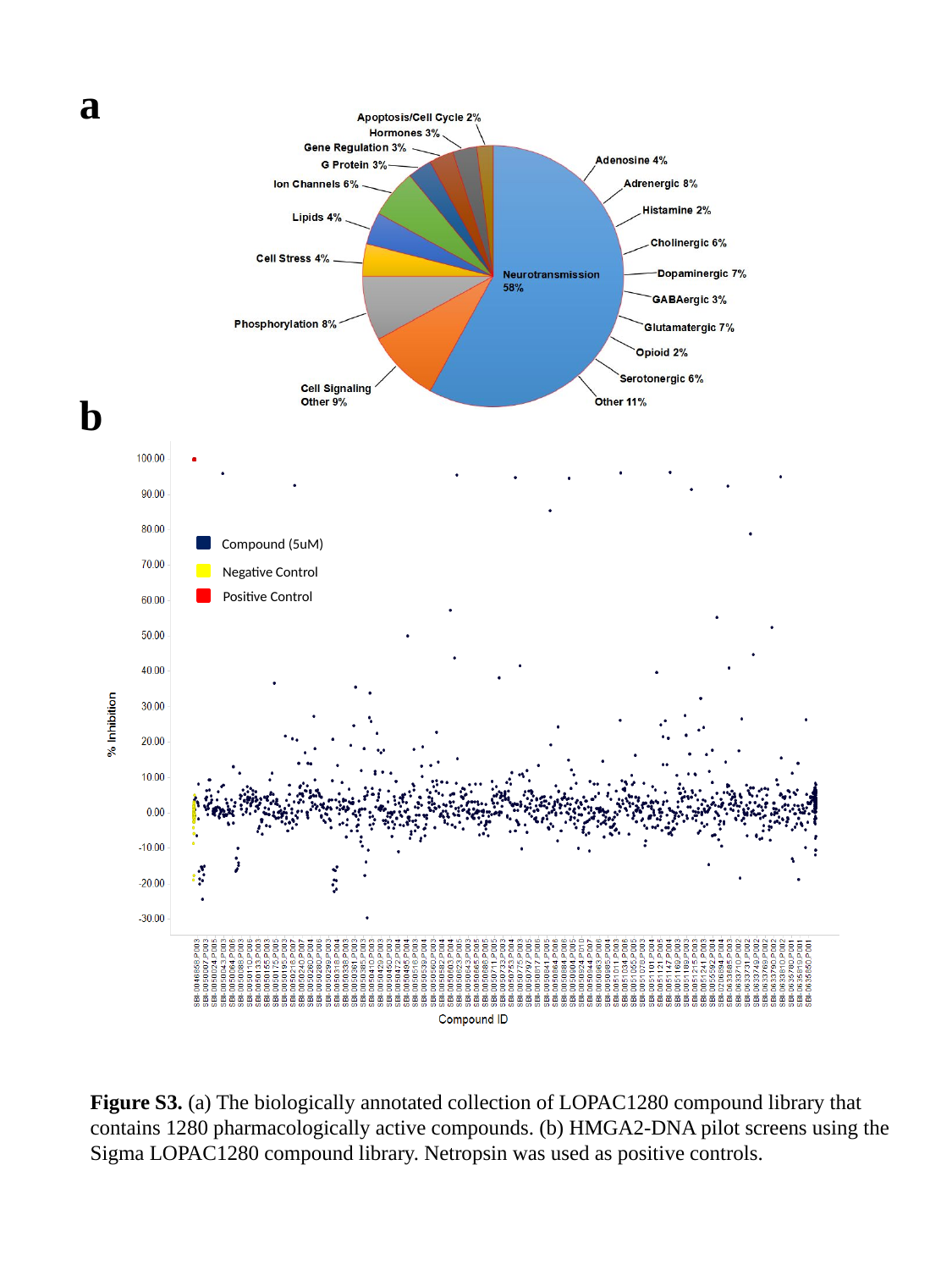

a
b
Compound (5uM)
Negative Control
Positive Control
Figure S3. (a) The biologically annotated collection of LOPAC1280 compound library that contains 1280 pharmacologically active compounds. (b) HMGA2-DNA pilot screens using the Sigma LOPAC1280 compound library. Netropsin was used as positive controls.

## Slide 5
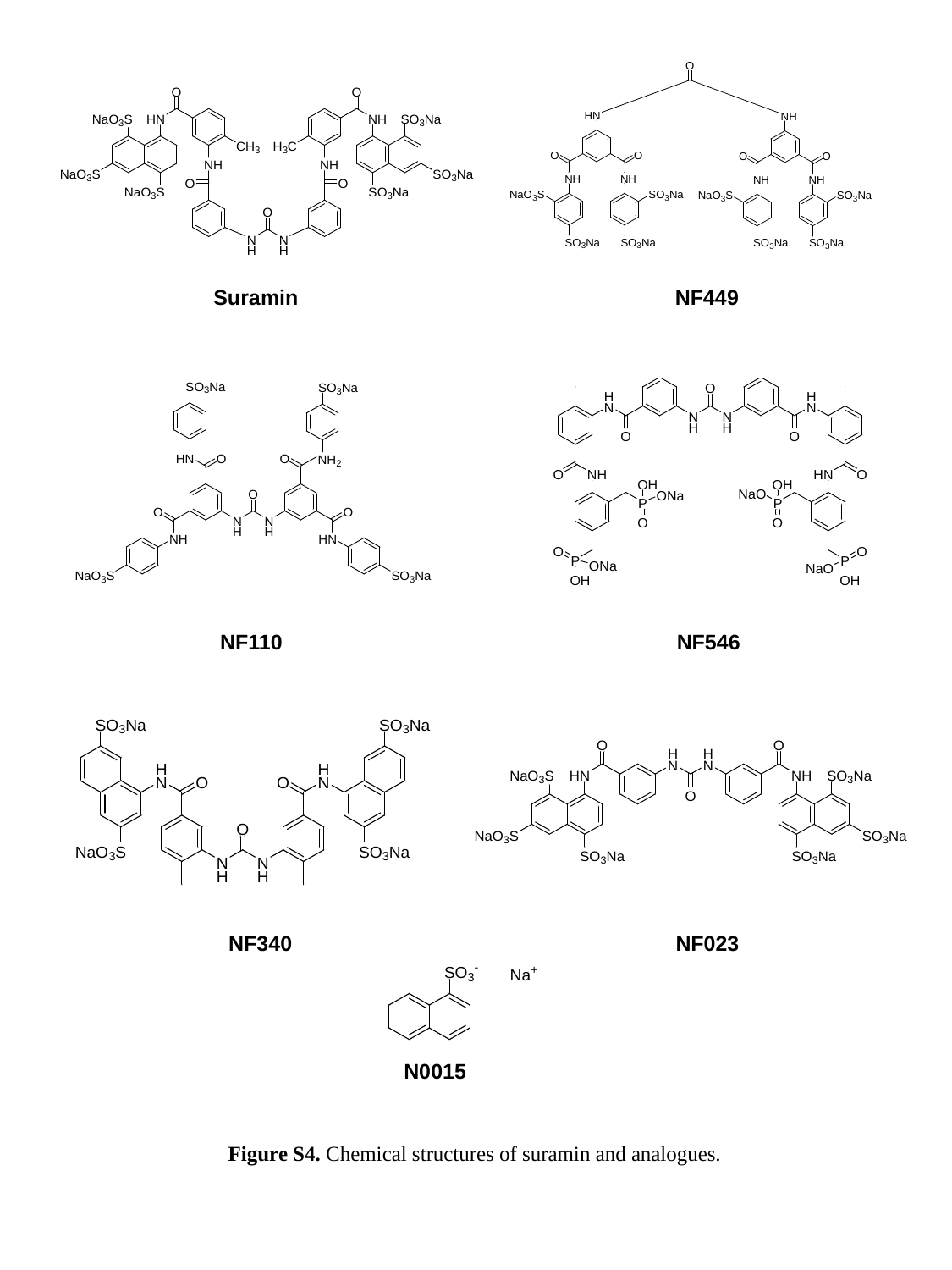

Suramin
NF449
NF110
NF546
NF023
NF340
N0015
Figure S4. Chemical structures of suramin and analogues.

## Slide 6
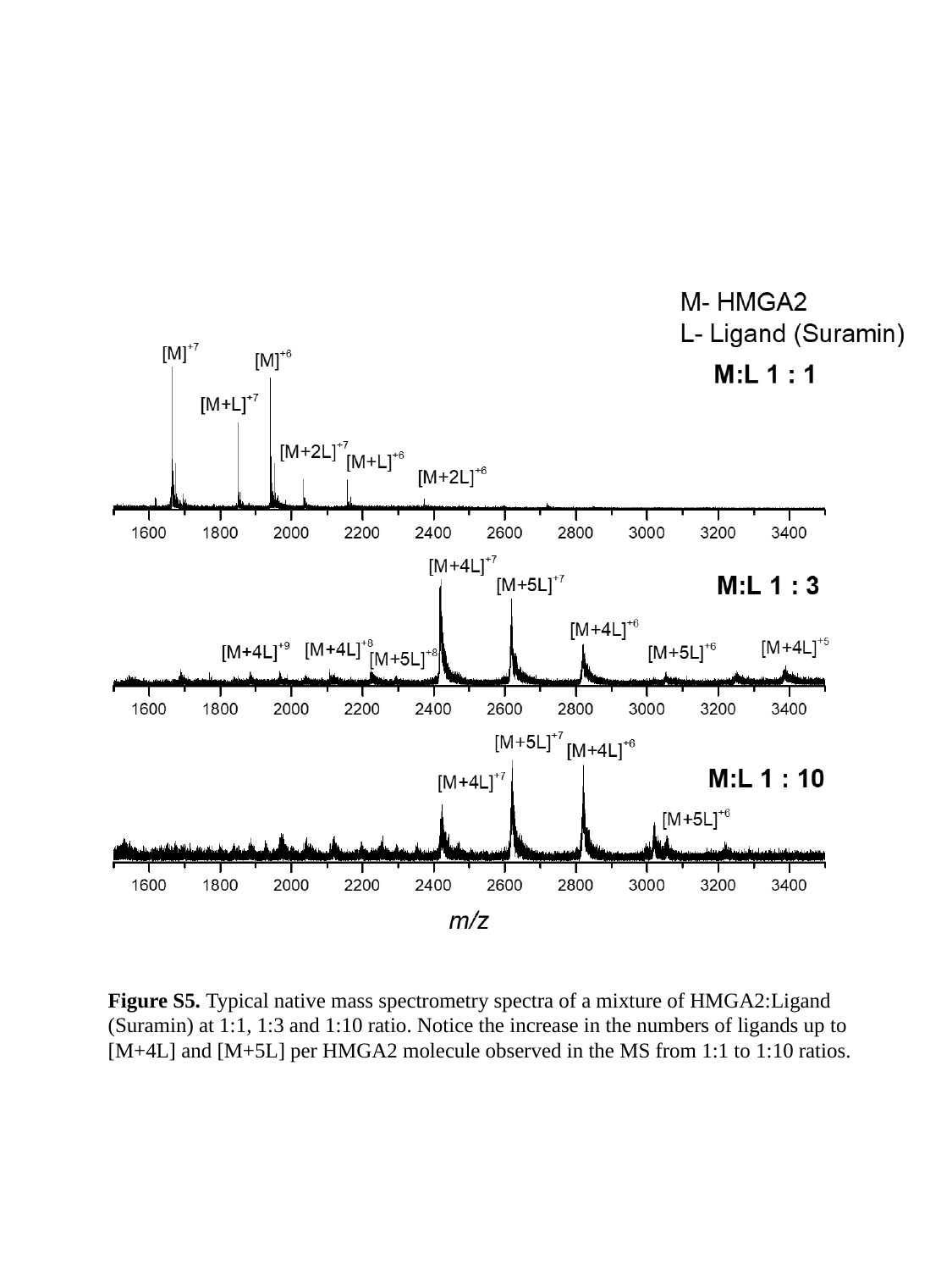

Figure S5. Typical native mass spectrometry spectra of a mixture of HMGA2:Ligand (Suramin) at 1:1, 1:3 and 1:10 ratio. Notice the increase in the numbers of ligands up to [M+4L] and [M+5L] per HMGA2 molecule observed in the MS from 1:1 to 1:10 ratios.

## Slide 7
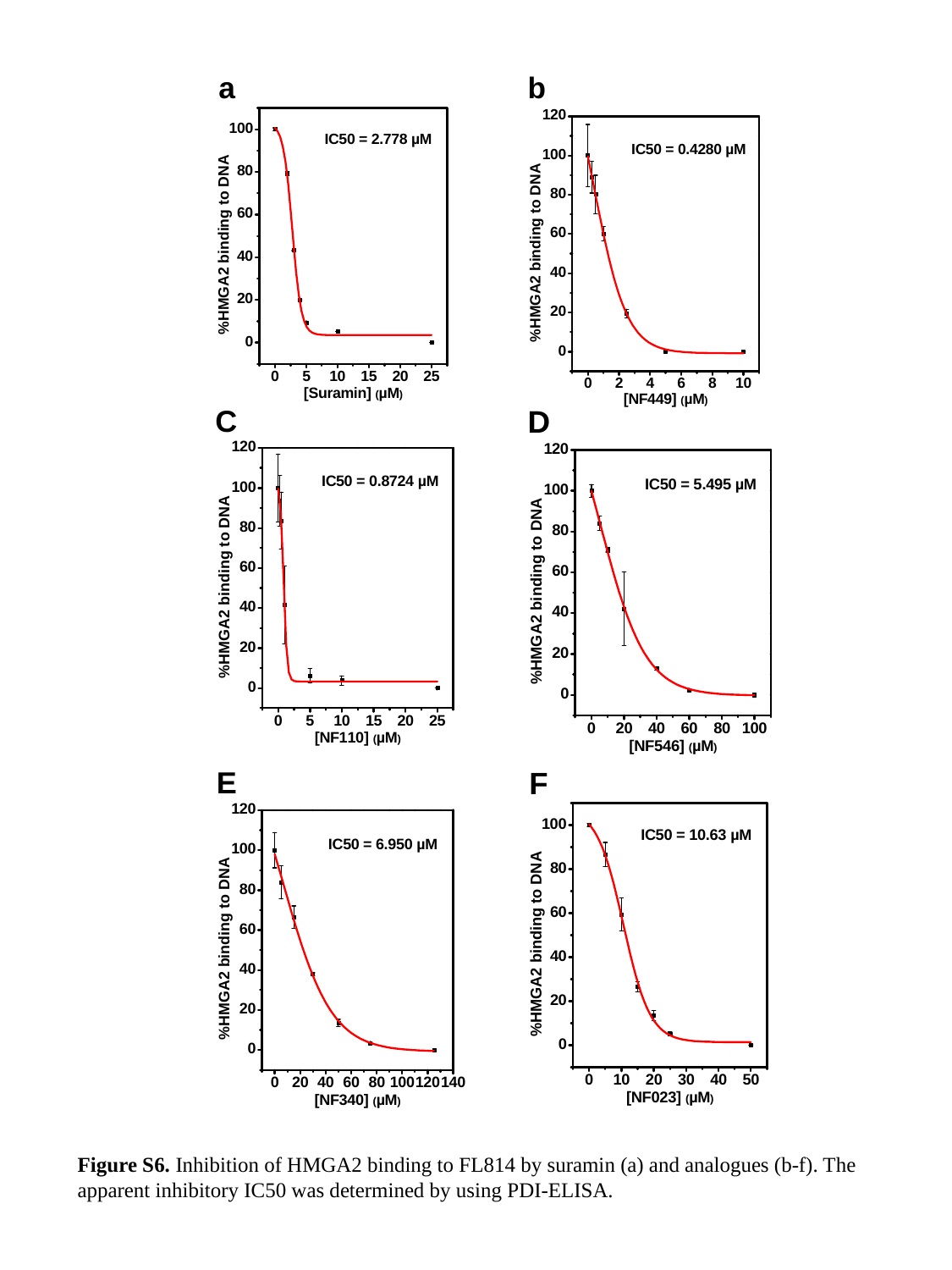

Figure S6. Inhibition of HMGA2 binding to FL814 by suramin (a) and analogues (b-f). The apparent inhibitory IC50 was determined by using PDI-ELISA.

## Slide 8
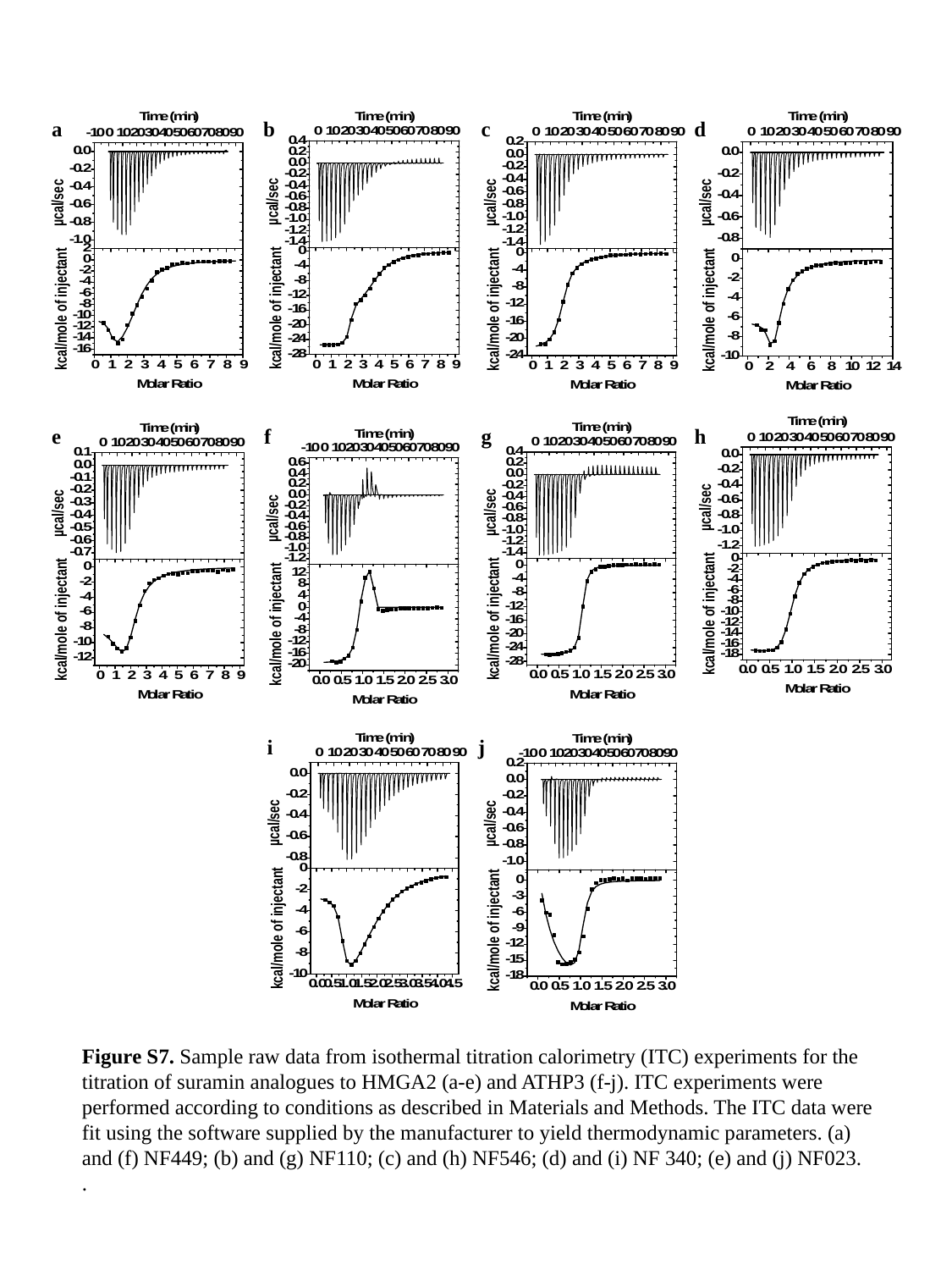

c
d
b
a
g
h
f
e
j
i
Figure S7. Sample raw data from isothermal titration calorimetry (ITC) experiments for the titration of suramin analogues to HMGA2 (a-e) and ATHP3 (f-j). ITC experiments were performed according to conditions as described in Materials and Methods. The ITC data were fit using the software supplied by the manufacturer to yield thermodynamic parameters. (a) and (f) NF449; (b) and (g) NF110; (c) and (h) NF546; (d) and (i) NF 340; (e) and (j) NF023.
.

## Slide 9
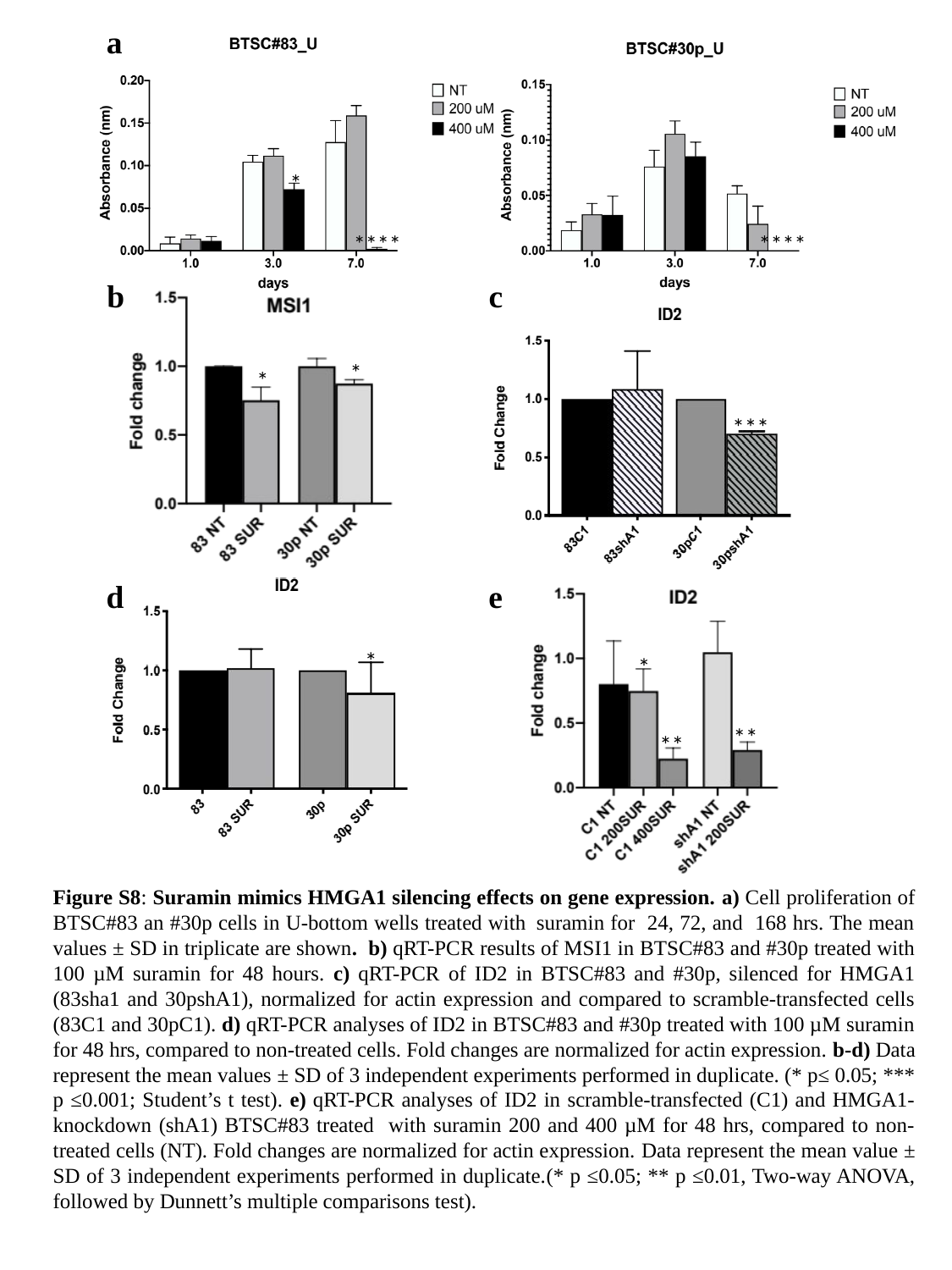

a
 *
****
****
*
*
***
*
*
**
**
b
c
d
e
Figure S8: Suramin mimics HMGA1 silencing effects on gene expression. a) Cell proliferation of BTSC#83 an #30p cells in U-bottom wells treated with  suramin for 24, 72, and 168 hrs. The mean values ± SD in triplicate are shown. b) qRT-PCR results of MSI1 in BTSC#83 and #30p treated with 100 µM suramin for 48 hours. c) qRT-PCR of ID2 in BTSC#83 and #30p, silenced for HMGA1 (83sha1 and 30pshA1), normalized for actin expression and compared to scramble-transfected cells (83C1 and 30pC1). d) qRT-PCR analyses of ID2 in BTSC#83 and #30p treated with 100 µM suramin for 48 hrs, compared to non-treated cells. Fold changes are normalized for actin expression. b-d) Data represent the mean values ± SD of 3 independent experiments performed in duplicate. (* p≤ 0.05; *** p ≤0.001; Student’s t test). e) qRT-PCR analyses of ID2 in scramble-transfected (C1) and HMGA1-knockdown (shA1) BTSC#83 treated with suramin 200 and 400 µM for 48 hrs, compared to non-treated cells (NT). Fold changes are normalized for actin expression. Data represent the mean value ± SD of 3 independent experiments performed in duplicate.(* p ≤0.05; ** p ≤0.01, Two-way ANOVA, followed by Dunnett’s multiple comparisons test).

## Slide 10
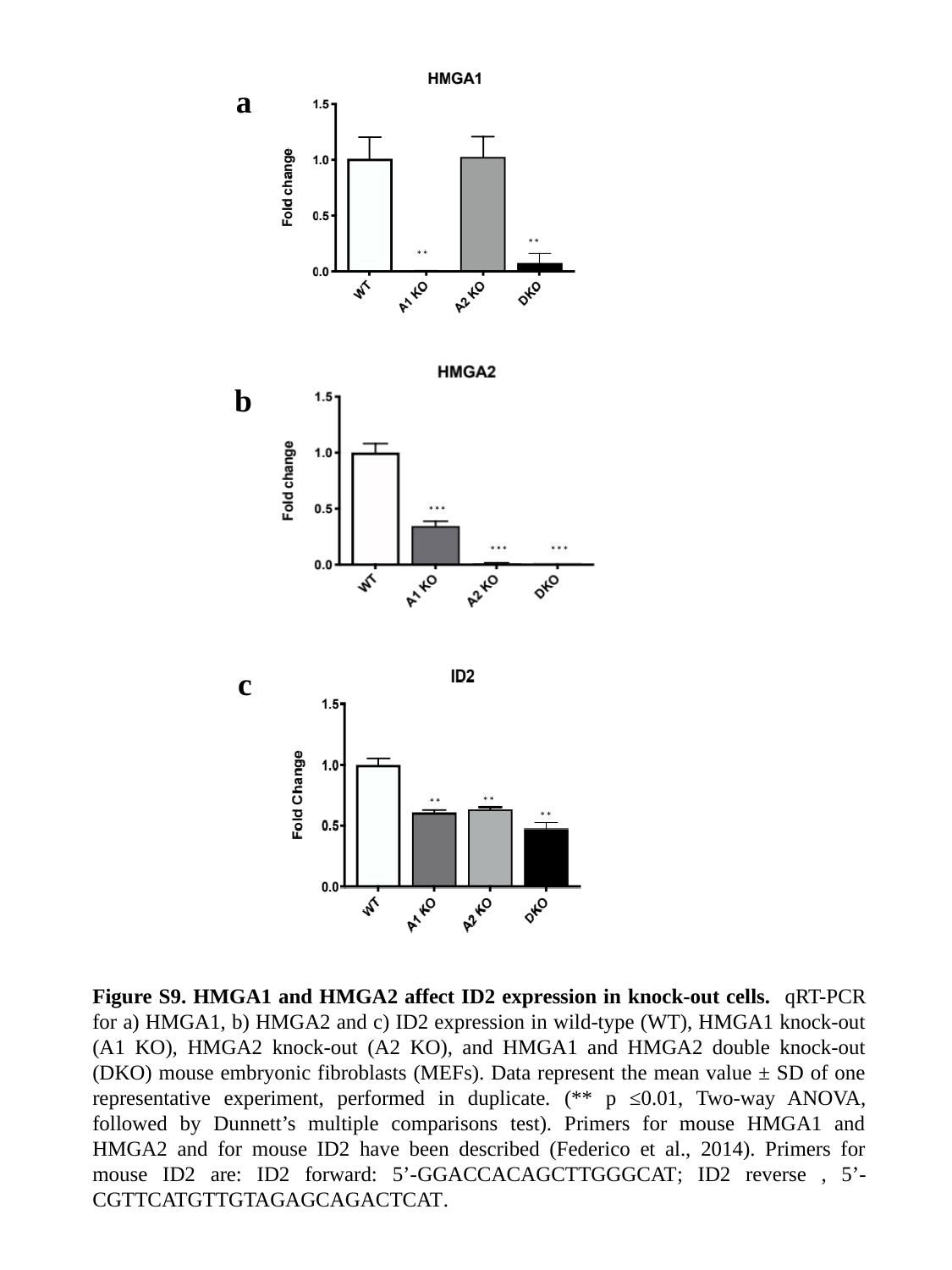

**
**
a
***
***
***
b
c
**
**
**
Figure S9. HMGA1 and HMGA2 affect ID2 expression in knock-out cells. qRT-PCR for a) HMGA1, b) HMGA2 and c) ID2 expression in wild-type (WT), HMGA1 knock-out (A1 KO), HMGA2 knock-out (A2 KO), and HMGA1 and HMGA2 double knock-out (DKO) mouse embryonic fibroblasts (MEFs). Data represent the mean value ± SD of one representative experiment, performed in duplicate. (** p ≤0.01, Two-way ANOVA, followed by Dunnett’s multiple comparisons test). Primers for mouse HMGA1 and HMGA2 and for mouse ID2 have been described (Federico et al., 2014). Primers for mouse ID2 are: ID2 forward: 5’-ggaccacagcttgggcat; ID2 reverse , 5’-cgttcatgttgtagagcagactcat.

## Slide 11
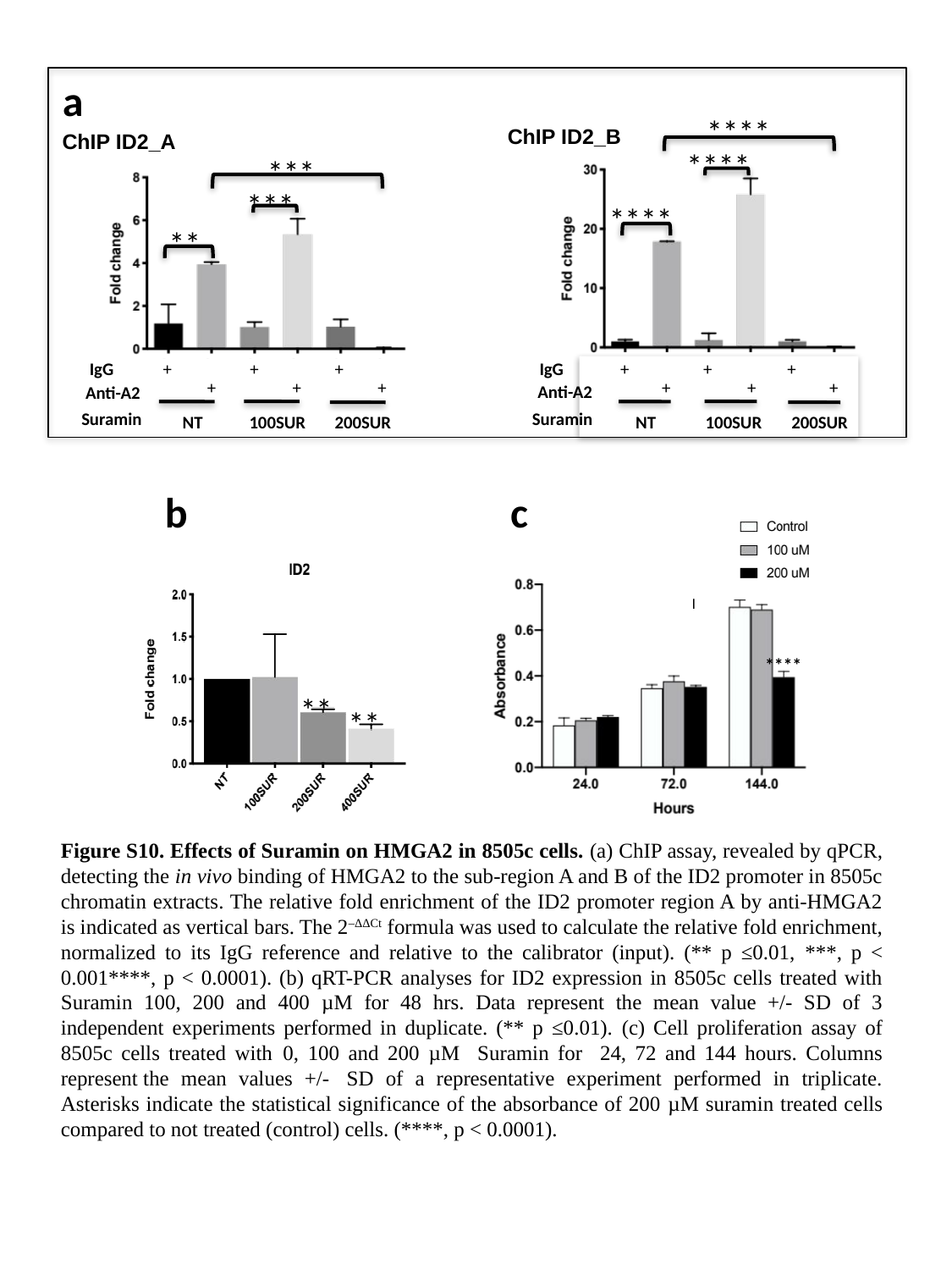

a
****
ChIP ID2_B
ChIP ID2_A
****
***
***
****
**
IgG
+
+
+
+
+
+
Anti-A2
Suramin
200SUR
NT
100SUR
IgG
+
+
+
+
+
+
Anti-A2
Suramin
200SUR
NT
100SUR
b
c
****
**
**
Figure S10. Effects of Suramin on HMGA2 in 8505c cells. (a) ChIP assay, revealed by qPCR, detecting the in vivo binding of HMGA2 to the sub-region A and B of the ID2 promoter in 8505c chromatin extracts. The relative fold enrichment of the ID2 promoter region A by anti-HMGA2 is indicated as vertical bars. The 2–ΔΔCt formula was used to calculate the relative fold enrichment, normalized to its IgG reference and relative to the calibrator (input). (** p ≤0.01, ***, p < 0.001****, p < 0.0001). (b) qRT-PCR analyses for ID2 expression in 8505c cells treated with Suramin 100, 200 and 400 µM for 48 hrs. Data represent the mean value +/- SD of 3 independent experiments performed in duplicate. (** p ≤0.01). (c) Cell proliferation assay of 8505c cells treated with  0, 100 and 200 µM Suramin for 24, 72 and 144 hours. Columns represent the mean values +/-  SD of a representative experiment performed in triplicate. Asterisks indicate the statistical significance of the absorbance of 200 µM suramin treated cells compared to not treated (control) cells. (****, p < 0.0001).

## Slide 12
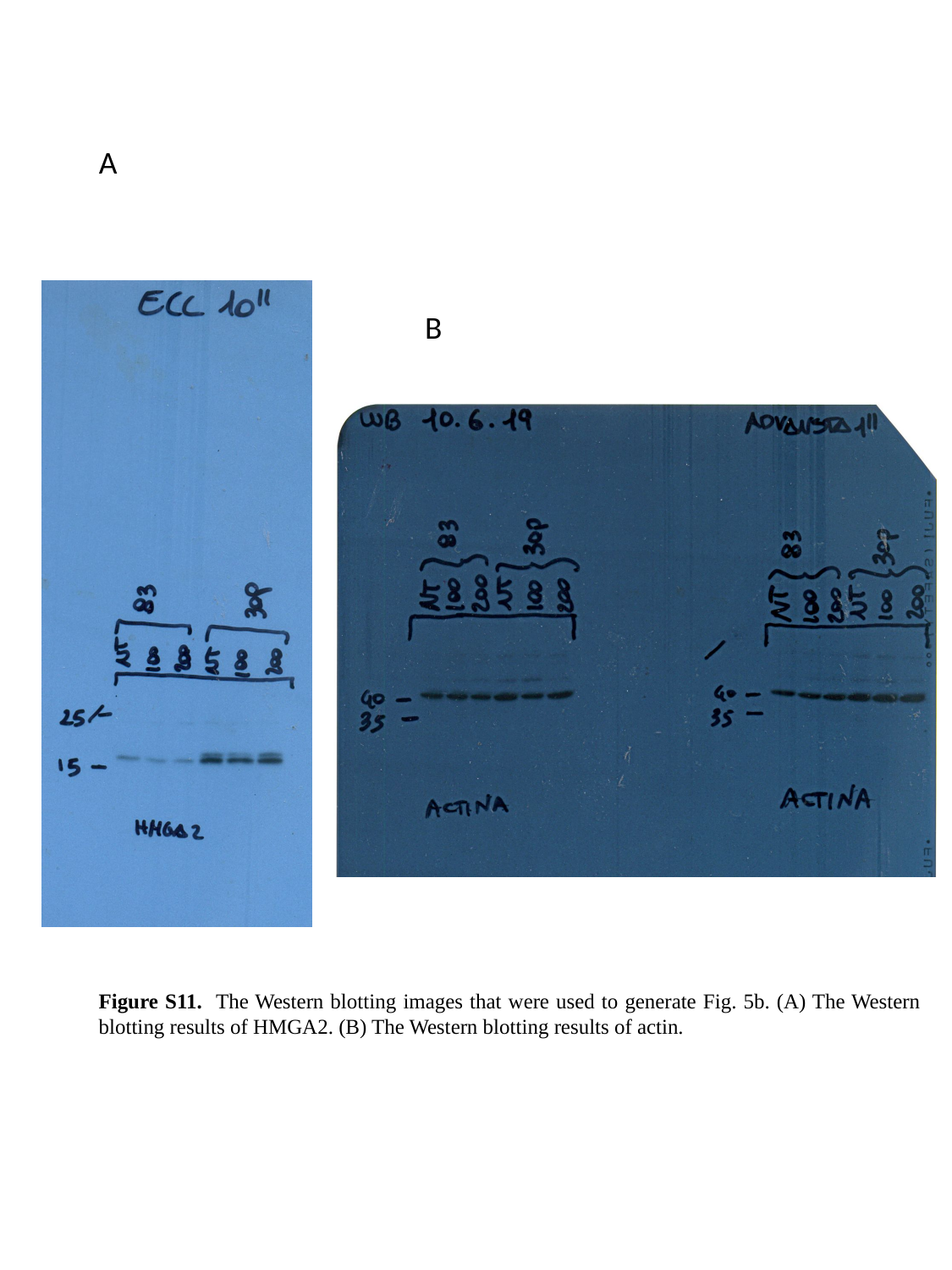

A
B
Figure S11. The Western blotting images that were used to generate Fig. 5b. (A) The Western blotting results of HMGA2. (B) The Western blotting results of actin.

## Slide 13
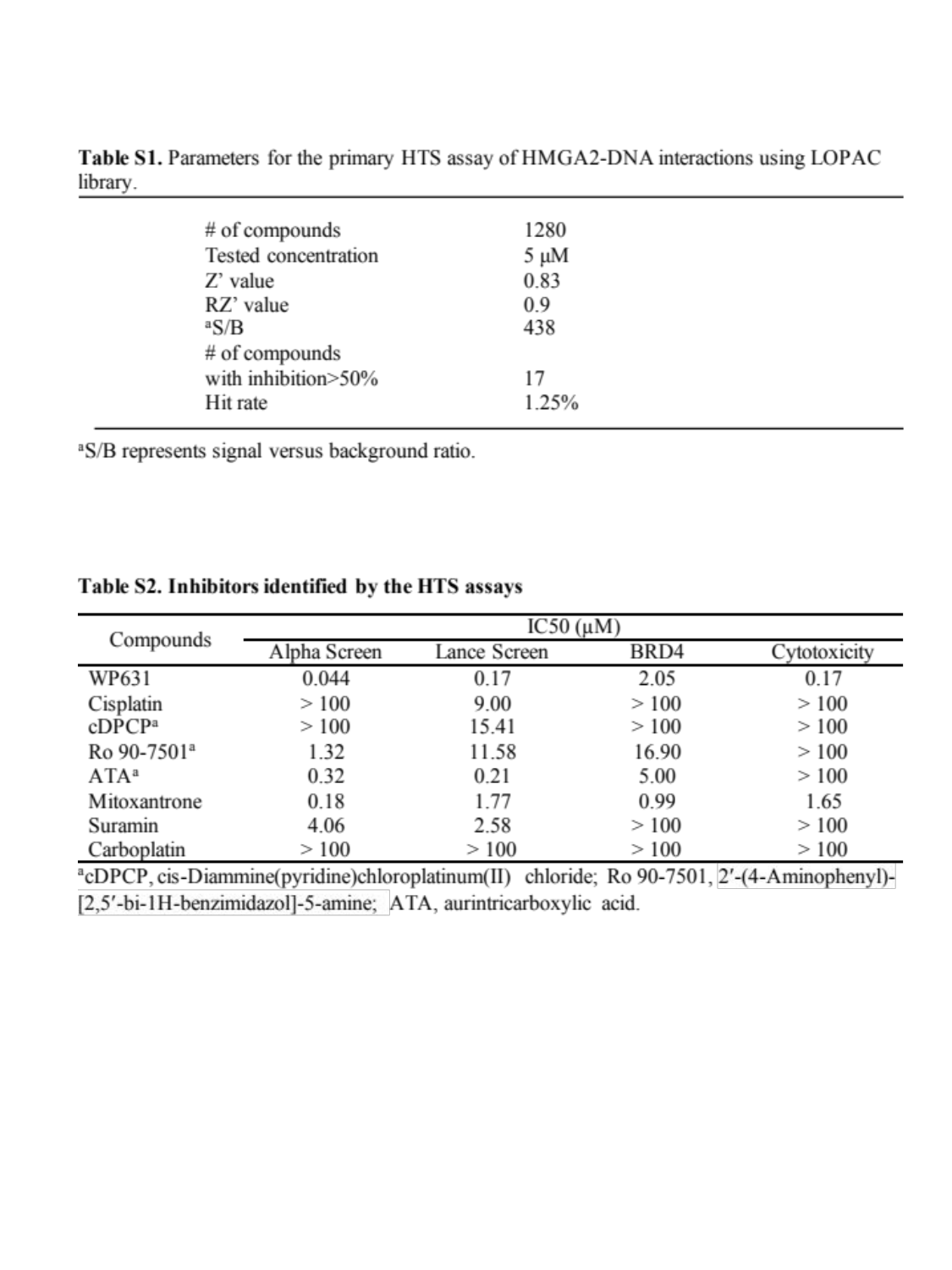

## Slide 14
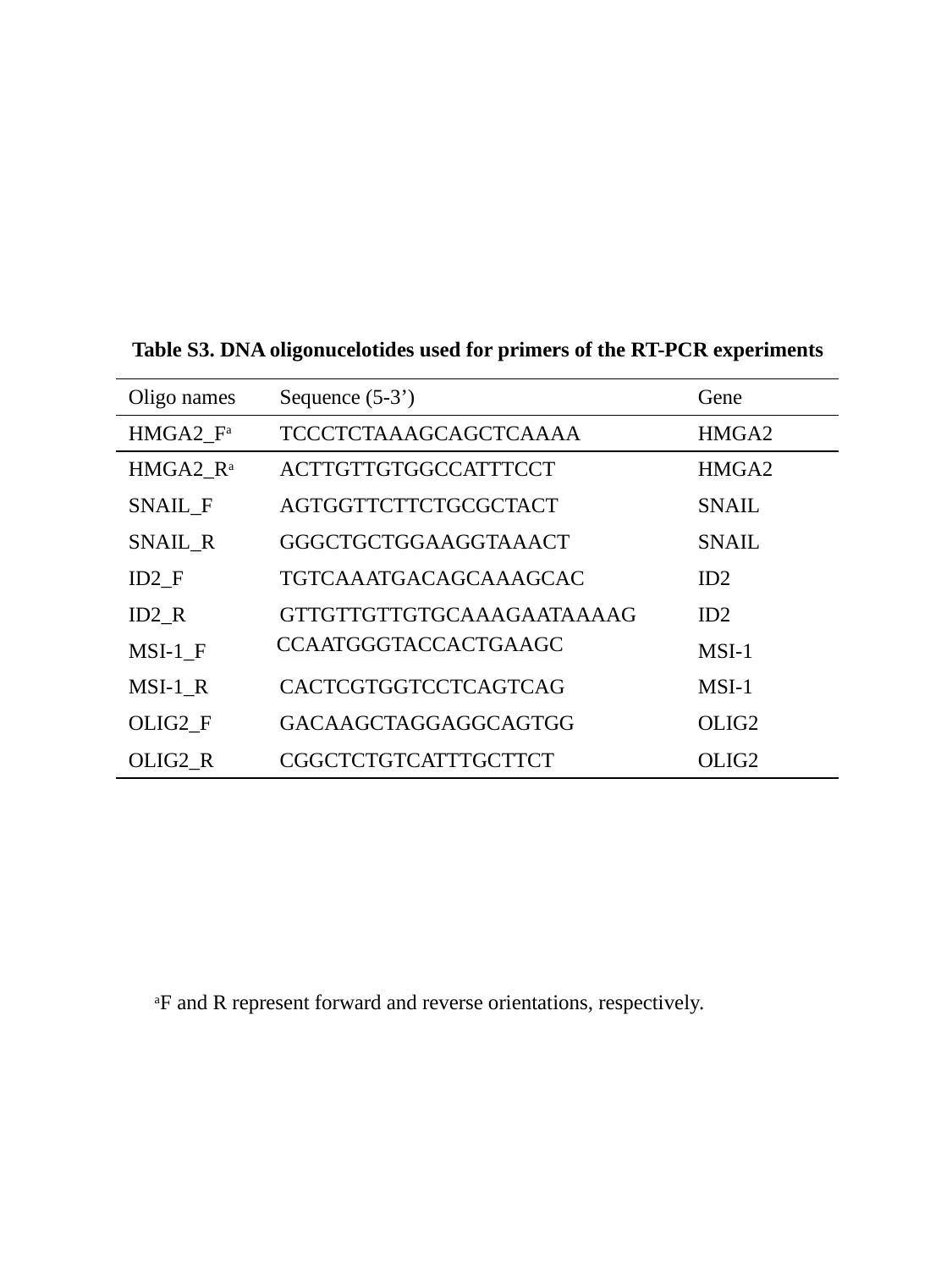

Table S3. DNA oligonucelotides used for primers of the RT-PCR experiments
| Oligo names | Sequence (5-3’) | Gene |
| --- | --- | --- |
| HMGA2\_Fa | TCCCTCTAAAGCAGCTCAAAA | HMGA2 |
| HMGA2\_Ra | ACTTGTTGTGGCCATTTCCT | HMGA2 |
| SNAIL\_F | AGTGGTTCTTCTGCGCTACT | SNAIL |
| SNAIL\_R | GGGCTGCTGGAAGGTAAACT | SNAIL |
| ID2\_F | tgtcaaatgacagcaaagcac | ID2 |
| ID2\_R | gttgttgttgtgcaaagaataaaag | ID2 |
| MSI-1\_F | CCAATGGGTACCACTGAAGC | MSI-1 |
| MSI-1\_R | CACTCGTGGTCCTCAGTCAG | MSI-1 |
| OLIG2\_F | GACAAGCTAGGAGGCAGTGG | OLIG2 |
| OLIG2\_R | CGGCTCTGTCATTTGCTTCT | OLIG2 |
aF and R represent forward and reverse orientations, respectively.
